# Prognostic value of plasma brain‐derived phosphorylated Tau

**DOI:** 10.1002/alz.71983

**Published:** 2026-11-13

**Authors:** Valentina Ghisays, Marisa N. Denkinger, Alpana Singh, Tainá M. Marques, Michael Malek‐Ahmadi, Kendall Van‐Keuren Jensen, Hillary D. Protas, Javad Sohankar, Dhruman D. Goradia, Vivek Devadas, Yinghua Chen, Shan Li, Jessica B. Langbaum, Michael W. Weiner, Eric M. Reiman, Yi Su, Nicholas J. Ashton, for the Alzheimer's Disease Neuroimaging Initiative

**Affiliations:** ^1^ Banner Alzheimer's Institute Phoenix Arizona USA; ^2^ Banner Sun Health Research Institute Sun City Arizona USA; ^3^ Translational Genomics Research Institute Phoenix Arizona USA; ^4^ University of California San Francisco San Francisco California USA; ^5^ Department of Psychiatry and Neurochemistry Institute of Neuroscience & Physiology Sahlgrenska Academy at the University of Gothenburg Mölndal Sweden

**Keywords:** Alzheimer's disease progression, amyloid PET progression, brain‐derived pTau217, plasma biomarkers, plasma phosphorylated tau

## Abstract

**INTRODUCTION:**

Plasma brain‐derived phosphorylated Tau217 (BD‐pTau217) may provide a more Alzheimer's disease (AD) ‐specific plasma tau measure than total pTau217, but its prognostic value is unclear.

**METHODS:**

We compared baseline plasma NULISAseq biomarkers for amyloid positron emission tomography (PET) progression (positivity: Centiloid ≥ 24.1; n = 175) and clinical progression to mild cognitive impairment or AD dementia (n = 508) in cognitively unimpaired Alzheimer's Disease Neuroimaging Initiative (ADNI) participants using Cox models and time‐dependent areas under the curve (tdAUCs).

**RESULTS:**

Over a median clinical follow‐up of 6 years (interquartile range [IQR], 3.0–7.3), BD‐pTau217 and total pTau217 had similar discrimination (full‐follow‐up AUC = 0.66 each; high‐versus‐low hazard ratios [HRs]; 95% confidence interval [CIs]: 2.8 [1.89–4.06] and 2.9 [1.96–4.35], respectively). Among initially amyloid‐negative participants, BD‐pTau217 better predicted progression to amyloid PET positivity at 2.5 years (tdAUC 0.82 vs. 0.69; HR = 10.54 [95% CI 2.3–48.1], *p* = 0.00015) and 4 years (tdAUC 0.77 vs. 0.64; HR = 7.03 [95% CI 1.9–25.6], *p* = 0.00055) compared to total pTau217.

**CONCLUSION:**

BD‐pTau217 better predicted near‐term progression to amyloid PET positivity than total pTau217, but showed no advantage for clinical progression, supporting its potential value as an early marker of AD pathological change.

## Highlights

- Brain‐derived phosphorylated Tau217 (BD‐pTau217) predicted amyloid positron emission tomography (PET )progression in cognitively uni, paired (CU) Alzheimer's Disease Neuroimaging Initiative (ADNI) participants.
- BD‐pTau217 outperformed total pTau217 at the Centiloid (CL) ≥ 24.1 threshold.
- BD‐pTau217 and total pTau217 performed similarly for clinical progression.
- Prognostic performance varied across amyloid PET thresholds.
- BD‐pTau217 may support an improved risk stratification in prevention trials.

## 1 BACKGROUND

The ability to estimate the presence of Alzheimer's disease (AD) pathology through blood‐based measures represents a major advancement in both AD research and, increasingly, clinical care. In symptomatic individuals, elevated levels of phosphorylated Tau217 (pTau217) in the blood strongly suggest that the presenting symptomatology is attributable to AD, as a strong indicator of neuritic amyloid plaque pathology. Conversely, low levels indicate that AD pathology is unlikely to be present at a clinically significant level, and that the observed cognitive deficits may be better explained by an alternative cause.^1^

It is consistently reported that the positive predictive value (PPV) of pTau217 is lower than its negative predictive value (NPV). This difference is partly attributable to lower disease prevalence in certain tested populations (e.g., cognitively unimpaired) but also partially reflects that elevated pTau217 levels can be occasionally observed due to other reasons, such as chronic kidney disease (CKD) or other medical conditions^2^. In these instances, only more severe cases are likely to elevate pTau217 sufficiently to shift an individual into a different classification group.^3^ It has been shown that in patients with amyotrophic lateral sclerosis (ALS), p‐tau levels, including pTau217, are increased in the blood^4^, ^5^ but not cerebrospinal fluid (CSF).^6^ This points to a potentially specific peripheral expression of p‐tau, localized, for example, to striated muscle tissue in ALS^4^ but also introduces uncertainty regarding other possible sources of circulating p‐tau if such tests are widely adopted in unselected, heterogeneous primary and secondary care populations.

Therefore, to mitigate potential peripheral contributions, whose frequency and impact remain uncertain, the use of brain‐derived phosphorylated Tau217 (BD‐pTau217) immunoassays is essential. Tau expressed in the periphery includes a 254‐amino acid insert encoded by exon 4a, commonly referred to as “big tau.” Because the sandwich immunoassay design of most pTau217 tests targets epitopes that span regions flanking exon 4a, these assays are not able to distinguish between peripheral pTau217 (if present) and pTau217 released from the central nervous system (CNS) in response to AD pathology. A BD‐pTau217 immunoassay can be designed using the same capture antibody targeting phosphorylated threonine 217, but with a detector antibody raised against the junction created when exon 4a is absent. In this configuration, the assay selectively recognizes CNS‐type tau and excludes “big tau” isoforms containing the exon 4a insert. To demonstrate this, Janelidze and colleagues compared different plasma pTau217 assay designs in ALS patients and found increases in total pTau217, but not BD‐pTau217.^6^ Therefore, with a more specific assay design, enhanced diagnostic performance of BD‐pTau217 for AD pathology has been reported ^7^, ^8^, ^9^ including comparisons with post‐mortem neuropathology.^10^ In AD, cross‐sectional studies suggest that BD‐pTau217 offers modest diagnostic advantages over total plasma pTau217 for identifying cerebral AD pathology.^7^

However, to date, there has not been an evaluation of the prognostic performance of BD‐pTau217 (and other BD‐pTau assays) compared to total pTau. In this study, we sought to address this knowledge gap by assessing the ability of plasma BD‐pTau217 to predict amyloid positron emission tomography (PET) and clinical progression in the Alzheimer's Disease Neuroimaging Initiative (ADNI) cohort.

**RESEARCH IN CONTEXT**

1. **Systematic review**: Plasma phosphorylated Tau217 (pTau217) biomarkers show strong cross‐sectional associations with amyloid and tau pathology and are increasingly used to support Alzheimer's disease diagnosis and trial screening. Recent assay designs selectively measuring brain‐derived pTau217 may reduce peripheral contributions to total pTau217 measurements, but their longitudinal prognostic value relative to total pTau217 remains less well established.
2. **Interpretation**: In cognitively unimpaired Alzheimer's Disease Neuroimaging Initiative (ADNI) participants, plasma brain‐derived pTau217 (BD‐pTau217) showed stronger prognostic performance than total pTau217 for progression from amyloid positron emission tomography (PET) ‐negative to amyloid PET positive status, particularly at the primary Centiloid (CL) ≥ 24.1 threshold. In contrast, BD‐pTau217 and total pTau217 showed more similar performance for predicting clinical progression to mild cognitive impairment (MCI) or dementia.
3. **Future directions**: These findings support further evaluation of BD‐pTau217 for biological risk stratification and prevention trial enrichment. Validation is needed in more diverse cohorts, over longer follow‐up intervals, and across clinical‐grade assay platforms.

## 2 METHODS

### 2.1 Study participants

Data were obtained from the ADNI cohort. Participants with available plasma NULISAseq CNS 120 panel biomarker measurements were included. Diagnostic classification at baseline followed ADNI criteria for CU, MCI, or AD dementia. Demographic characteristics including age, sex, education, and average amyloid PET Centiloid (CL) values are shown in Table 1. For longitudinal analyses, participants were required to have baseline NULISAseq measurement and longitudinal amyloid PET or clinical diagnostic follow‐up. Prognostic analyses focused on baseline CU participants: amyloid PET progression analyses were restricted to CU participants without amyloid PET positivity at baseline, whereas clinical progression analyses followed CU participants for progression to MCI or AD dementia. All analyses were performed with publicly available de‐identified data from ADNI1/GO/2/3 participants (Figure 1).

**TABLE 1:** Baseline demographic and clinical characteristics of the plasma NULISAseq cohort

Parameter	CU (N = 529)	MCI (N = 675)	AD dementia (N = 223)	Total (N = 1427)	p‐Value
Age, years	72.9 ± 6.3	72.4 ± 7.6	74.7 ± 8.2	73.0 ± 7.3	<0.001
Female, n (%)	310 (58.6%)	276 (40.9%)	89 (39.9%)	675 (47.3%)	<0.001
Education, years	16.7 ± 2.5	16.1 ± 2.7	15.5 ± 2.9	16.2 ± 2.7	<0.001
Amyloid PET CL	20.2 ± 37.0	46.7 ± 53.1	93.9 ± 44.0	43.1 ± 52.0	<0.001

*Note*: Baseline demographic and clinical characteristics of ADNI participants with plasma NULISAseq measurements, overall and by baseline diagnosis group. P‐values reflect overall group comparisons across CU, MCI, and AD dementia using ANOVA for continuous variables and chi‐square test for sex. Values are mean ± SD unless otherwise indicated.

Abbreviations: AD dementia, Alzheimer's disease dementia; ADNI, Alzheimer's Disease Neuroimaging Initiative; ANOVA, analysis of variance; CL, Centiloid; CU, cognitively unimpaired; MCI, mild cognitive impairment; PET, positron emission tomography; SD, standard deviation.

**FIGURE 1:**
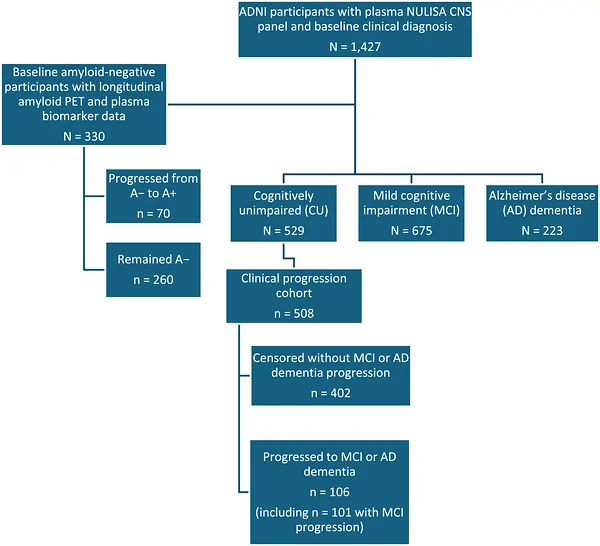
Study design and analytic cohorts. Flow diagram of participants from the ADNI with available plasma NULISAseq CNS panel measurements and an available baseline clinical diagnosis (N = 1,427). Participants were categorized by baseline clinical diagnosis (CU, MCI, or Alzheimer's disease dementia). Clinical progression analyses included baseline CU participants with longitudinal diagnostic follow‐up (n = 508). Amyloid PET analyses were drawn from participants with longitudinal amyloid PET and plasma biomarker data; the primary amyloid PET progression analysis was restricted to baseline CU participants who were amyloid‐negative at baseline at the primary CL ≥ 24.1 threshold and had longitudinal amyloid PET follow‐up (n = 175). Subsets were included in longitudinal analyses of (1) amyloid PET progression among participants with baseline amyloid‐negative (A−) status and follow‐up PET imaging and (2) clinical diagnostic progression among baseline CU participants with longitudinal diagnostic data. The threshold‐specific sensitivity cohorts included N = 166 at CL ≥ 18 and N = 185 at CL ≥ 37. Sample sizes differ across analytic cohorts due to availability of longitudinal imaging, baseline amyloid status, clinical diagnosis, and clinical follow‐up. ADNI, Alzheimer's Disease Neuroimaging Initiative; CL, Centiloid; CNS, central nervous system; CU, cognitively unimpaired; MCI, mild cognitive impairment; PET, positron emission tomography;

### 2.2 Image analysis

Amyloid PET data ([18F]‐florbetapir PET scans) from baseline and follow‐up were preprocessed with an in‐house PET unified pipeline (PUP) ^11^, ^12^, ^13^ using corresponding baseline T1‐weighted structural magnetic resonance imaging (MRI) scans for anatomical labeling in individual space with FreeSurfer v7 (http://surfer.nmr.mgh.harvard.edu/).^14^ Briefly, for semi‐quantitative Aβ burden, mean cortical standard uptake value ratios (mcSUVRs) were extracted and averaged across the set of FreeSurfer‐defined cortical regions of interest including frontal, parietal, temporal, and precuneus.^11^, ^13^ We converted the mcSUVRs calculated with a cerebellar reference region^15^ to CL values.^16^

Amyloid PET positivity (A+) was defined using a primary threshold of CL ≥ 24.1, selected a priori because it discriminates neuropathologically confirmed none or sparse versus moderate to frequent plaques in autopsy‐confirmed cases.^17^ For cross‐sectional analyses, amyloid PET status was determined with the closest visit associated with the NULISAseq measurement and for longitudinal assessments, follow‐up scans were used to assess progression from amyloid‐negative (A−) to A+. In sensitivity analyses, alternative literature‐supported thresholds for A+ were additionally evaluated (Centiloid ≥ 18 and ≥ 37).^18^, ^19^, ^20^ These analyses were performed to assess robustness of biomarker performance across commonly used definitions of A+.

### 2.3 Biomarker measurements

Plasma biomarkers were measured at Michael T. Zuendel Biomarker Laboratory at Banner Sun Health Research Institute on an ARGO HT platform (Alamar Biosciences) using the NULISAseq^TM^ CNS Disease Panel.^21^, ^22^, ^23^ We used the NULISA Protein Quantification (NPQ) values on the log2 scale with intra‐and‐inter plate normalization. The highly multiplexed panel measures neurodegenerative disease‐related biomarkers in plasma or CSF with ultra‐broad dynamic range that can quantify extremely low concentrations of biomarkers like pTau217 in the attomolar range along with highly concentrated proteins from the same 250 µl sample.

Brain‐derived and total versions of phosphorylated tau biomarkers were evaluated, with BD‐pTau217 designated as the primary biomarker of interest based on prior evidence of strong diagnostic performance. The primary comparison focused on BD‐pTau217 versus total pTau217. BD‐pTau181 was included as an additional brain‐derived phosphorylated tau comparator in the primary amyloid progression table, while other pTau species, glial fibrillary acidic protein (GFAP), and pTau/Aβ ratios were evaluated in secondary sensitivity analyses. Given that NULISAseq NPQ values are reported on a log2 scale, pTau/Aβ42 and Aβ40/Aβ42 measures were calculated by subtracting the denominator NPQ from the numerator NPQ. These measures represent log2 NPQ‐derived relative ratio scores and should not be interpreted as absolute concentration ratios.

### 2.4 Longitudinal outcomes

Longitudinal outcomes included amyloid PET progression and clinical diagnostic progression. For amyloid progression analyses, baseline was defined as the earliest available amyloid PET assessment with a matched baseline plasma NULISAseq measurement. Analyses were restricted to CU participants who were A− at baseline, as defined in Section 2.2, and who had longitudinal amyloid PET follow‐up. Participants were followed to progression to A+ status or censored at their last available PET visit if they remained A−. *Time‐to‐event* was calculated from the baseline A− PET visit to the first A+ PET visit or last follow‐up. For sensitivity analyses, amyloid progression was also evaluated using alternative thresholds of CL ≥ 18 and CL ≥ 37.^18^, ^19^, ^20^ Baseline biomarker measurements were matched to the baseline amyloid PET assessment using the closest available plasma sample within a 365‐day window. The broader baseline A− cohort with longitudinal amyloid PET and plasma biomarker data comprised 330 participants across diagnostic groups. The primary amyloid progression analysis was restricted a priori to 175 participants who were cognitively unimpaired and amyloid‐negative at baseline and had longitudinal florbetapir PET follow‐up. These restrictions reflected the study questions and availability of the required longitudinal outcomes rather than selection based on plasma biomarker values. Sensitivity cohorts included 166 participants at CL ≥ 18 and 185 at CL ≥ 37.

For clinical progression analyses, baseline was defined as the plasma NULISAseq collection date. Analyses included participants who were CU at plasma sampling, had no recorded MCI or AD dementia diagnosis on or before plasma collection, and had at least one subsequent diagnostic assessment. Participants were followed from plasma collection until the first qualifying progression event or were censored at their last available diagnostic assessment. Clinical progression was evaluated as progression from CU to MCI or AD dementia and, separately, from CU to MCI. Two participants with conflicting CU and MCI diagnoses recorded on the same date were excluded. *Time‐to‐event* was defined as the time interval between baseline plasma biomarker collection and the first recorded diagnosis of MCI or AD dementia, or censoring at the last available diagnostic assessment. Of the 1,427 ADNI participants with plasma NULISAseq measurements and an available baseline clinical diagnosis, 529 were cognitively unimpaired at baseline. Clinical progression analyses included 508 of these participants with longitudinal diagnostic follow‐up.

### 2.5 Statistical analysis

Longitudinal prognostic performance of the plasma biomarkers was assessed using complementary time‐to‐event and discrimination approaches. Cox proportional hazards (CPH) models were used to estimate hazard ratios (HRs) for amyloid and clinical progression associated with baseline biomarker levels. For binary comparisons, biomarker groups were defined using Youden index‐derived cutoffs estimated from receiver operating characteristic (ROC) analyses. These cutoffs were used to define “high” and “low” biomarker groups for Kaplan–Meier (KM), log‐rank, and Cox model comparisons. For each biomarker and Centiloid threshold, a single Youden cutoff was estimated using conversion status over the full available follow‐up and was then applied unchanged in the 2.5‐ and 4‐year horizon‐restricted Cox and log‐rank analyses.

KM curves were used to visualize risk separation between biomarker‐defined high and low groups over time. For amyloid progression analyses, Cox models, log‐rank tests, C‐indices, and tdAUCs were evaluated at prespecified 2.5‐ and 4‐year horizons by censoring follow‐up at each horizon. For clinical progression analyses, Cox models, log‐rank tests, C‐indices, and full‐follow‐up AUCs were evaluated across the full available follow‐up, while tdAUCs were calculated at 2.5 and 4 years to summarize short‐term discrimination. Analyses focused on relative discrimination and ranking performance rather than absolute risk prediction.

We compared BD and total pTau biomarkers using paired analyses where applicable. Statistical significance was evaluated using two‐sided tests. In secondary adjusted Cox models, continuous biomarker values were standardized. Clinical progression models were adjusted for age, sex, education, and apolipoprotein E (*APOE) ε4* copy number; amyloid PET progression models were adjusted for age, sex, and *APOE ε4* copy number to evaluate whether associations were independent of these demographic and genetic risk factors. Analyses were performed in R (version 4.4.3) using the survival, riskRegression, timeROC, and pROC packages.

## 3 RESULTS

### 3.1 Baseline characteristics

We included 1,427 participants with plasma NULISAseq biomarker measurements with a mean age 73.0 (SD ±7.3 years). Baseline age, sex distribution, education, and amyloid PET CL values differed across diagnostic groups (all *p* < 0.001; Table 1). AD participants were older on average and had higher amyloid PET CL values than CU and MCI participants, while the CU group had a higher proportion of women. Prognostic analyses focused on baseline CU participants, including CU participants with longitudinal clinical follow‐up for clinical progression analyses and CU participants who were A− at baseline with longitudinal amyloid PET follow‐up for amyloid PET progression analyses.

### 3.2 Amyloid PET progression

Among CU participants with available baseline amyloid PET, 186 were A− and 76 were A+ at the primary CL ≥ 24.1 threshold. Baseline plasma BD‐pTau217 levels were higher in CU A+ than CU A− participants (median 11.9 [interquartile range {IQR} 11.6–12.3] vs. 11.0 [10.7–11.2] NPQ), as were total pTau217 levels (median 11.8 [11.3–12.1] vs. 10.6 [10.3–11.0] NPQ). For longitudinal amyloid progression analyses, 175 baseline CU A− participants with longitudinal amyloid PET follow‐up were included. At the primary amyloid positivity threshold (CL ≥ 24.1), BD‐pTau217 showed the strongest prognostic performance, whereas total pTau217 and BD‐pTau181 showed weaker and non‐significant associations (Table 2). BD‐pTau217 showed the highest time‐dependent discrimination across follow‐up, with prespecified 2.5‐ and 4‐year estimates reported in Table 2 and the broader tdAUC trajectory is shown in Figure 2A. This pattern was also apparent in the KM analyses, in which BD‐pTau217 showed clearer risk separation than total pTau217 over follow‐up (Figure 2B,C). At this primary threshold, most ratio measures and alternative pTau species, including BD‐pTau231 and pTau231‐based measures, showed weaker or non‐significant associations, supporting BD‐pTau217 as the strongest overall predictor.

**TABLE 2:** Prognostic performance of plasma biomarkers for amyloid PET progression (A− to A+) in baseline cognitively unimpaired participants at the primary amyloid positivity threshold (Centiloid ≥ 24.1)

Marker	tdAUC (2.5 years)	HR (95% CI) High vs. low	Log‐rank *p*	C‐Index	tdAUC (4 years)	HR (95% CI) High vs. low	Log‐rank *p*	C‐Index
BD‐pTau217	0.82	10.54 (2.31–48.10)	0.00015	0.76	0.77	7.03 (1.94–25.56)	0.00055	0.73
pTau217	0.69	2.15 (0.47–9.79)	0.31267	0.57	0.64	1.42 (0.39–5.17)	0.58945	0.54
BD‐pTau181	0.66	2.33 (0.63–8.60)	0.19180	0.59	0.60	1.75 (0.54–5.69)	0.34484	0.57

*Note*: Discrimination is summarized using tdAUC at prespecified 2.5‐ and 4‐year horizons. HRs and C‐indices were derived from Cox proportional hazards models comparing high versus low biomarker groups within each follow‐up horizon. Log‐rank *p*‐values compare KM curves for the same high versus low biomarker groups. High versus low biomarker groups were defined using Youden index cutoffs derived from overall amyloid progression status across the full follow‐up period.

Abbreviations: BD‐pTau, brain‐derived phosphorylated Tau; CI, confidence interval; HR, hazard ratio; KM, Kaplan–Meier; PET, positron emission tomography; pTau, phosphorylated Tau; tdAUC, time‐dependent area under the curve.

**FIGURE 2:**
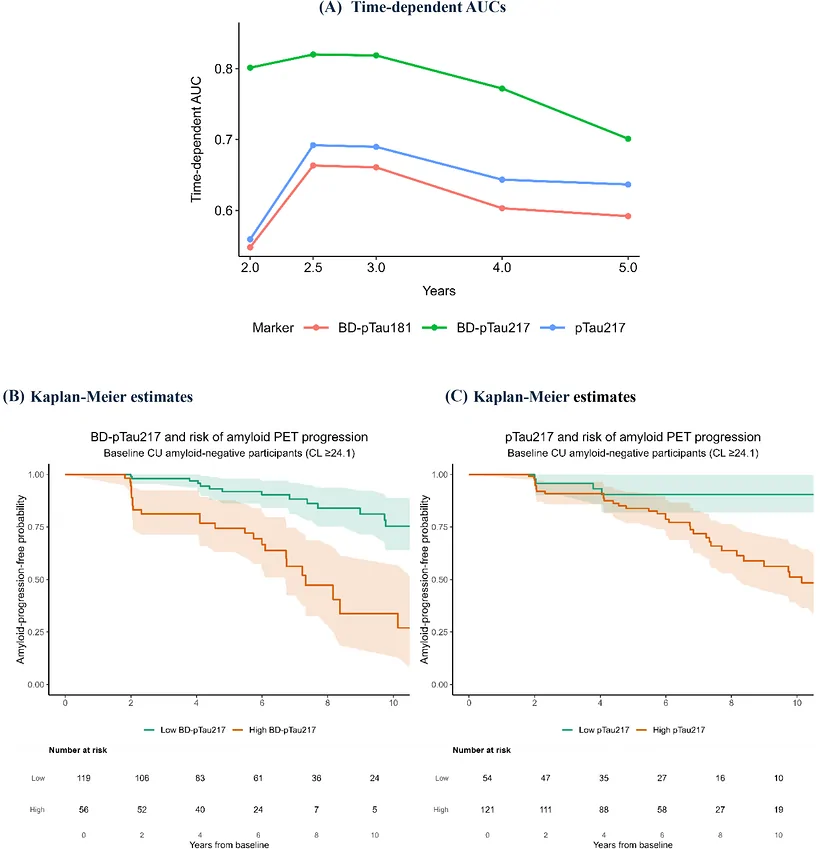
Prognostic performance of plasma pTau biomarkers for amyloid PET progression. (A) Time‐dependent AUC trajectories for prediction of amyloid PET progression (A− to A+) among baseline CU participants at the primary amyloid positivity threshold (CL ≥ 24.1). Table 2 reports the prespecified 2.5‐ and 4‐year estimates. BD‐pTau217 demonstrated consistently higher discrimination than total pTau217 and BD‐pTau181. KM estimates of amyloid‐progression‐free probability according to high versus low baseline BD‐pTau217 (B) and total pTau217 (C), defined using Youden index cutoffs. Curves reflect cumulative risk separation over the full available follow‐up period among CU participants who were A− at baseline (CL ≥ 24.1) with shaded 95% confidence bands. AUC, area under the curve; BD‐pTau217, brain‐derived phosphorylated Tau217; CL, Centiloid; CU, cognitively unimpaired; KM, Kaplan–Meier; pTau, phosphorylated Tau; PET, positron emission tomography

At 2.5 years (N = 175; converters = 12), elevated baseline BD‐pTau217 was strongly associated with amyloid PET progression (HR 10.54, 95% CI 2.3–48.1; log‐rank *p* = 0.00015; tdAUC 0.82). In contrast, total pTau217 and BD‐pTau181 were not significantly associated with progression and showed substantially lower discrimination. At 4 years (converters = 13), BD‐pTau217 remained the strongest predictor (HR 7.03, 95% CI 1.9–25.6; log‐rank *p* = 0.00055; tdAUC 0.77), while total pTau217 and BD‐pTau181 again demonstrated weak and non‐significant associations (Table 2). These results suggest that prognostic performance at the primary threshold was strongest for BD‐pTau217 rather than reflecting a uniform signal across pTau measures.

Across sensitivity analyses, biomarker performance varied according to the amyloid positivity threshold used (Tables S1‐S2). At the lower threshold (CL ≥ 18), discrimination was generally weaker and less consistent across single biomarkers, although selected pTau/Aβ42 ratio measures, including BD‐pTau217/Aβ42 and pTau217/Aβ42, showed stronger associations with amyloid progression compared with their corresponding single‐marker measures.

At the higher threshold (CL ≥ 37), several biomarkers and ratio measures demonstrated improved prognostic performance, including total pTau217, BD‐pTau181, BD‐pTau231, and corresponding pTau/Aβ42 ratio measures, consistent with increasing signal at more advanced stages of amyloid burden. Notably, at both CL ≥ 18 and CL ≥ 37, estimates at the 2.5‐ and 4‐year horizons were identical (although the corresponding tdAUCs varied slightly) because all amyloid conversion events observed within the 4‐year horizon occurred within the first 2.5 years (e.g., CL ≥ 18: 14 events by 2.5 years and 4 years; CL ≥ 37: 13 events by 2.5 years and 4 years), whereas at the primary threshold (CL ≥ 24.1), additional events occurring after 2.5 years resulted in differing estimates between time horizons. Overall, BD‐pTau217 demonstrated the highest observed time‐dependent discrimination at the primary CL ≥ 24.1 threshold.

In secondary adjusted Cox models, baseline BD‐pTau217 remained associated with amyloid PET progression after adjustment for age, sex, and *APOE ε4* copy number at both 2.5 years (HR 1.43 per SD, 95% CI 1.07–1.92; *p* = 0.0155) and 4 years (HR 1.39 per SD, 95% CI 1.03–1.86; *p* = 0.029). Total pTau217 was not significantly associated with amyloid PET progression at 2.5 years (HR 1.37 per SD, 95% CI 0.92–2.04; *p* = 0.12) or 4 years (HR 1.28 per SD, 95% CI 0.85–1.93; *p* = 0.23).

### 3.3 Clinical progression

Among the 508 baseline CU participants included, 106 progressed to MCI or AD dementia during a median follow‐up of 6.0 years (IQR 3.0–7.3). When restricting progression to MCI only, 101 participants progressed and 407 had no recorded MCI event. Five participants progressed directly to AD dementia without an intervening recorded MCI diagnosis.

Participants with high total pTau217 levels had a 2.9‐fold increased risk of progression compared with those in the low group (HR 2.9, 95% CI 1.96–4.35; log‐rank *p* < 0.001), with progression observed in 32.4% of the high group versus 12.8% of the low group. BD‐pTau217 showed comparable risk estimates (HR 2.8, 95% CI 1.89–4.06; high group 35.8% vs. low group 14.6% progressed). Similar risk estimates were observed for BD‐pTau181 (HR 2.7, 95% CI 1.81–4.05). Full‐follow‐up AUCs were similar across markers (0.65–0.66), and concordance indices ranged from 0.62 to 0.63. Time‐dependent discrimination at 2.5 and 4 years was also modest and overlapped across markers (tdAUCs approximately 0.59–0.63) (Table 3, Figure 3). KM analyses showed separation for both BD‐pTau217 and total pTau217, but overall differences between markers were smaller than for amyloid PET progression (Figure 3B‐C).

**TABLE 3:** Prognostic performance of plasma pTau biomarkers for clinical progression from cognitively unimpaired to MCI or AD dementia

Outcome	Marker	tdAUC (2.5 years)	tdAUC (4 years)	AUC full follow‐up	HR (95% CI) High vs. low	Log‐rank *p*	C‐Index
CU→MCI/AD	pTau217	0.60	0.62	0.66	2.9 (1.96–4.35)	<0.001	0.63
CU→MCI/AD	BD‐pTau217	0.59	0.62	0.66	2.8 (1.89–4.06)	<0.001	0.62
CU→MCI/AD	BD‐pTau181	0.61	0.63	0.65	2.7 (1.81–4.05)	<0.001	0.63
CU→MCI	pTau217	0.60	0.62	0.66	2.95 (1.96–4.43)	<0.001	0.63
CU→MCI	BD‐pTau217	0.59	0.62	0.66	2.7 (1.85–4.04)	<0.001	0.62
CU→MCI	BD‐pTau181	0.61	0.63	0.65	2.75 (1.82–4.16)	<0.001	0.63

*Note*: The tdAUCs summarize discrimination at prespecified 2.5‐ and 4‐year horizons. Full‐follow‐up AUC summarizes continuous biomarker discrimination for overall clinical progression status across available follow‐up. HRs, log‐rank *p*‐values, and C‐indices are derived from full‐follow‐up Cox and KM analyses comparing high versus low biomarker groups. High versus low groups were defined using Youden index cutoffs derived from overall clinical progression status. CU→MCI and CU→MCI/AD yielded similar tdAUC estimates at the 2.5‐ and 4‐year horizons because nearly all early clinical progression events captured by these horizons were transitions from CU to MCI.

Abbreviations: AD, Alzheimer's disease; AUC, area under the curve; BD‐pTau217, brain‐derived phosphorylated Tau217; CU, cognitively unimpaired; KM, Kaplan–Meier; MCI, mild cognitive impairment; MCI, mild cognitive impairment; pTau, phosphorylated Tau; tdAUC, time‐dependent area under the curve.

**FIGURE 3:**
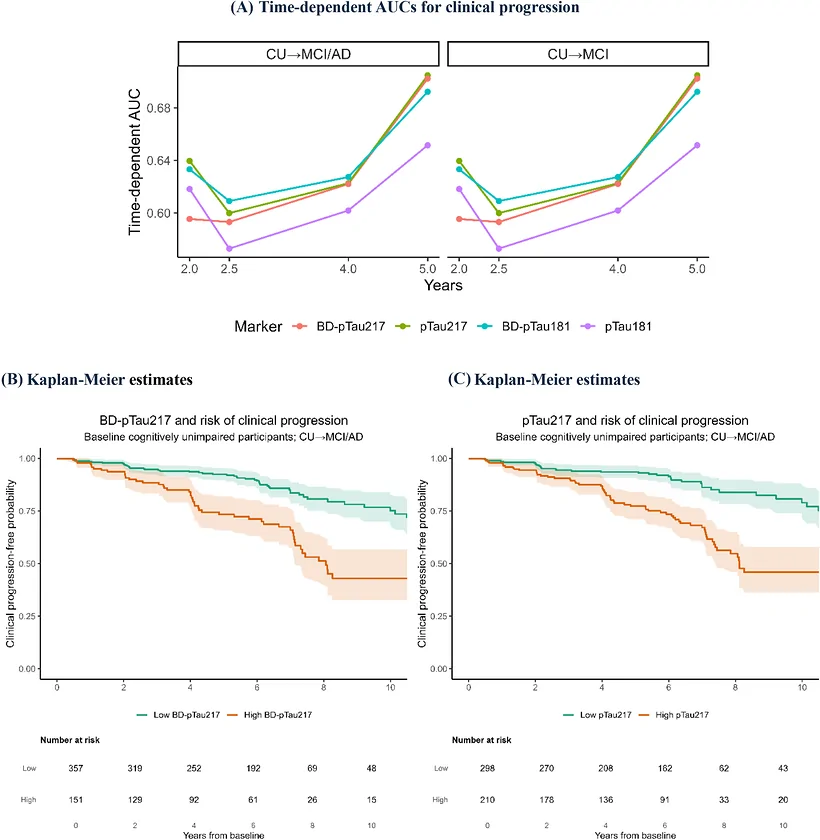
Prognostic performance of plasma pTau biomarkers for clinical progression. (A) Time‐dependent discrimination (tdAUC) for prediction of progression from CU status to MCI (CU→MCI) and to CU→MCI/AD across prespecified follow‐up horizons using baseline plasma biomarkers. Discrimination was modest and similar across biomarkers. KM estimates of clinical progression‐free probabilities according to baseline BD‐pTau217 level (B) and total pTau217 level (C), dichotomized using a Youden index cutoff among baseline CU participants with shaded 95% confidence bands. Higher biomarker levels were associated with greater cumulative risk of clinical progression over the full available follow‐up period. AD, Alzheimer's disease; BD‐pTau217, brain‐derived phosphorylated Tau217; CU, cognitively unimpaired; KM, Kaplan–Meier; MCI, mild cognitive impairment; tdAUC, time‐dependent area under the curve

Results were similar when we restricted progression to MCI alone, where high total pTau217 was associated with a 2.95‐fold increased risk of progression (95% CI 1.96–4.43), with progression rates of 31% in the high group versus 12.1% in the low group. BD‐pTau217 again had comparable performance (HR 2.7, 95% CI 1.85–4.04; progression rates 33.8% versus 14.0% in high and low groups, respectively), again with overlapping discrimination metrics across biomarkers (Table 3). Across models, discrimination for clinical progression was modest but consistent.

In secondary adjusted Cox models, for clinical progression to MCI or dementia (N = 508; 106 events) after adjustment for age, sex, education, and *APOE ε4* copy number, baseline BD‐pTau217 remained associated with progression (HR 1.39 per SD, 95% CI 1.18–1.64; *p* < 0.001; C‐index, 0.683), as did total pTau217 (HR 1.39 per SD, 95% CI 1.16–1.67; *p* < 0.001; C‐index, 0.684). Results were similar for progression restricted to MCI (N = 508; 101 events).

## 4 DISCUSSION

In this study, we evaluated the prognostic performance of brain‐derived and total plasma pTau biomarkers for amyloid PET and clinical progression in cognitively unimpaired ADNI participants with plasma NULISAseq measurements. We found that BD‐pTau217 demonstrated stronger prognostic value for amyloid PET progression, particularly at the primary A+ threshold of CL ≥ 24.1. At this threshold, BD‐pTau217 was the only primary marker to show strong and statistically significant prediction at both 2.5‐and 4‐year progression, whereas total pTau217 and BD‐pTau181 showed weaker and non‐significant associations. Sensitivity analyses across CL ≥ 18 and CL ≥ 37 further suggested that biomarker performance varies by amyloid threshold, with broader pTau and pTau/Aβ42 ratio^24^ signals emerging most clearly with alternative thresholds.

In contrast to findings for amyloid PET progression, differences between brain‐derived and total plasma pTau measures for prediction of clinical progression were modest, with both assay designs showing similar discrimination for progression from cognitively unimpaired status to MCI or dementia. Exploratory analyses further indicated that incorporation of plasma Aβ42 into biomarker ratios^24^ did not materially improve prediction beyond pTau217 alone for clinical outcomes. These findings suggest that while plasma pTau217 measures are associated with future clinical change, improvements in biological specificity achieved through brain‐derived measurements appear to have greater relevance for predicting underlying pathological progression than for short‐term clinical transitions.

Prior studies of plasma pTau biomarkers have largely focused on cross‐sectional discrimination of amyloid and tau pathology, consistently demonstrating better performance of plasma pTau217 to discriminate between the presence or absence of amyloid plaques and the diagnosis of AD compared with blood biomarkers.^6^, ^22^, ^25^, ^26^ Recent work has additionally suggested that selectively measuring brain‐derived fractions of plasma pTau may improve diagnostic performance.^7^, ^8^, ^9^, ^10^ Our results extend these observations by demonstrating that BD‐pTau217 provides stronger prognostic information for future amyloid accumulation than total plasma pTau217, particularly at the clinically relevant CL ≥ 24.1 threshold. Although brain‐derived and total pTau217 capture overlapping biological signal, the stronger and more consistent discrimination observed for BD‐pTau217 suggests that isolating brain‐origin tau species may enhance sensitivity to active amyloid‐related neurobiological change by reducing non‐specific signal from the periphery.

In analyses focused on amyloid PET progression, the relative performance of biomarkers depended on the amyloid positivity threshold used. At the primary CL ≥ 24.1 threshold, a neuropathologically validated cutoff in autopsy confirmed cases,^17^ BD‐pTau217 clearly outperformed total pTau217 and BD‐pTau181, which showed weak and non‐significant associations with progression. At the lower CL ≥ 18 threshold,^18^ single‐marker discrimination was weaker and less consistent, while selected pTau/Aβ42 ratio measures^24^, ^26^, including BD‐pTau217/Aβ42 and pTau217/Aβ42, showed stronger associations than their corresponding single‐marker measures.^27^, ^28^ This supports the notion that Aβ peptides in plasma may play an important role in the earliest phase of cerebral amyloid accumulation, despite challenges in robustness.^29^ At the higher CL ≥ 37 threshold,^19^ a broader set of biomarkers became informative, including total pTau217, BD‐pTau181, BD‐pTau231, and several pTau/Aβ42 ratio measures. This threshold‐dependent pattern suggests that different plasma biomarker combinations may be informative at different stages of amyloid accumulation, while BD‐pTau217 remains the most robust single‐marker predictor at the primary clinically relevant threshold.

Across clinical endpoints, discrimination for progression from CU to MCI or MCI/AD was modest and largely overlapping across biomarkers. BD‐pTau217, total pTau217, and BD‐pTau181 showed similar risk estimates and concordance values (HRs, C‐indices, and tdAUC), indicating that brain‐derived measurements did not clearly improve prediction of clinical progression. In contrast to amyloid PET progression, where BD‐pTau217 showed more pronounced advantage at the primary amyloid threshold, differences between brain‐derived and total pTau measures were small for clinical outcomes. The weaker separation for clinical outcomes is consistent with clinical progression being influenced by multiple factors beyond amyloid and plasma tau burden, including downstream neurodegeneration, vascular factors, comorbid pathology, and individual resilience. Furthermore, because incident MCI is etiologically heterogeneous and some events may not reflect AD as the primary cause, this endpoint definition may have attenuated the differences between an AD‐specific biomarker such as BD‐pTau217 and total pTau217.

The stronger prognostic performance observed for amyloid PET progression compared with clinical progression likely reflects the closer biological relationship between plasma pTau biomarkers and underlying AD pathology than with downstream clinical symptoms, as our group has recently shown comparing BD p‐tau217 against neuropathology.^10^ Cognitive decline results from a combination of amyloid and tau pathology but also neurodegeneration, vascular factors, comorbid pathology (e.g., TDP‐43), and individual resilience, making clinical progression inherently more heterogeneous and difficult to predict. In contrast, progression from A− to A+ status more directly reflects ongoing AD pathological processes, which plasma pTau biomarkers are thought to capture. These findings suggest that plasma pTau measurements, particularly BD‐pTau217, may be valuable for identifying cognitively unimpaired, A− individuals at elevated risk of near‐term amyloid progression, including those who may be candidates for primary prevention trials aimed at preventing progression of AD pathology.

Several considerations should be noted for the interpretation of these findings. First, plasma biomarkers were measured using the NULISAseq research platform, which provides highly sensitive, multiplexed relative quantification but differs from emerging in‐vitro diagnostic platforms intended for routine clinical use. Therefore, the absolute performance metrics in this study may not directly translate to the clinical assays, underscoring the need to further validate across independent cohorts and assay platforms.^7^, ^9^, ^10^ Second, clinical progression endpoints are influenced by multiple pathological and non‐pathological factors beyond amyloid and tau, which likely contributes to the moderate discrimination observed for the clinical outcomes. Third, the number of amyloid progression events was limited, particularly in horizon‐specific and threshold‐specific analyses, which may reduce the precision of some estimates. Lastly, we note that this was a single‐cohort, hypothesis generating analysis in a highly selected research sample. ADNI is a predominantly White and highly educated cohort (mean education, 16.2 years overall and 16.7 years among CU participants), detailed medical comorbidity burden was not included into the present models. Temporal alignment of the plasma measures and longitudinal PET or clinical follow‐up further restricted the analytic subset. More importantly, external validation in larger population‐based and clinically heterogeneous cohorts with greater racial, ethnic, socioeconomic, educational, and comorbidity diverse datasets will be necessary to determine biomarker performance across populations, assay platforms, and follow‐up intervals.

To summarize, plasma BD‐pTau217 has shown an added advantage in cross‐sectional prediction of AD pathology,^7^, ^8^, ^9^, ^10^ however, in this study, we demonstrated that BD‐pTau217 showed the highest observed time‐dependent discrimination at the primary CL ≥ 24.1 threshold for amyloid PET progression among cognitively unimpaired participants. At the primary ≥ 24.1 CL threshold, BD‐pTau217 clearly outperformed both pTau217 and BD‐pTau181, whereas differences between brain‐derived and total tau measures were smaller for predicting clinical progression. Sensitivity analyses indicated that biomarker performance varied across amyloid positivity thresholds, with selected pTau/Aβ42 ratio measures and additional pTau species becoming more informative at alternative cut points. Collectively, these preliminary findings support the use of BD‐pTau217 for risk stratification in prevention trials targeting the earliest detectable stages of Alzheimer's disease. Future studies should evaluate its performance in more diverse populations, over longer follow‐up periods, and across multiple assay platforms to facilitate clinical implementation.

## AUTHOR CONTRIBUTIONS

Nicholas J. Ashton and Valentina Ghisays conceived and designed the study. Marisa N. Denkinger, Alpana Singh, and Tainá M. Marques performed the biomarker measurements. Valentina Ghisays and Marisa N. Denkinger curated and processed the data. Valentina Ghisays performed the statistical analyses. Nicholas J. Ashton and Valentina Ghisays interpreted the findings. Valentina Ghisays and Nicholas J. Ashton drafted the manuscript. Nicholas J. Ashton supervised the study. All authors critically reviewed the manuscript and approved the final version.

## FUNDING

Data collection and sharing for ADNI were funded by the National Institute on Aging, National Insitute of Neurological Disorders and Stroke, and the National Institutes of Health grant U19 AG024904.

## CONFLICT OF INTEREST STATEMENT

The following authors have conflict of interests to disclose. M.W.W. serves on the Editorial Board for the Journal for Prevention of Alzheimer's Disease (JPAD) and served on the Editorial Board for Alzheimer's & Dementia from 2005‐2025. He has served on Advisory Boards for Acumen Pharmaceutical, Alzheon, Inc., Amsterdam UMC; MIRIADE, Cerecin, Merck Sharp & Dohme Corp., NC Registry for Brain Health, ProMIS Neurosciences, Inc., REGEnLIFE, ADNI Scientific Advisory Board leadership and UCSF Committee on Human Research membership, UCSF Inclusion, Diversity, Equity & Awareness Committee and ADNI Diversity Task Force roles. Institutional support from NIH grant 2 U19 AG024904.16, Kevin and Connie Shanahan, and the Veterans Administration. He also serves on the USC ACTC grant which receives funding from Eisai. He has provided consulting to Acadia Pharmaceuticals, Acumen Pharmaceuticals, Alzeca, Alzheon, Inc., Anven, ALZpath, Boxer Capital, LLC, Cerecin, Inc., Clario, Dementia Society of Japan, Dolby Family Ventures, Eisai, GLG Consulting, Guidepoint, Health and Wellness Partners, Indiana University, IXICO, LCN Consulting, MEDA Corp., Merck Sharp & Dohme Corp., Duke U.; NC Registry for Brain Health, NovoNordisk, Owkin France, ProMIS Neurosciences, Prova Education, Quantum Leap Health, REGEnLIFE, Sai MedPartners, T3D Therapeutics, Danaher, Medspoke, Splink Inc., Mitre, CervoMed, BrightFocus Foundation, U. Penn, University of Southern California (USC), and WebMD. He has acted as a speaker/lecturer for China Association for Alzheimer's Disease (CAAD) and Taipei Medical University, as well as a speaker/lecturer with academic travel funding provided by: AD/PD Congress, Amsterdam UMC, Banner Health, Cleveland Clinic, CTAD Congress, Foundation of Learning; Gates Ventures, Health Society (Japan), Kenes International, U. Madison Wisconsin, U. Penn, INSPIRE Project; U. Toulouse, Japan Society for Dementia Research, Korean Dementia Society, Merck Sharp & Dohme Corp., National Center for Geriatrics and Gerontology (NCGG; Japan), University of Madison Wisconsin, University of Southern California (USC), and Stead Impact Ventures. He holds stock with Alzeca, ALZPath, Inc., and Anven, and stock options with Alzheon, Inc. J.B.L. is supported by relevant grants R01 AG058468, R01 AG069453, P30AG072980, R01AG063954, R01AG086363, R33AG070604, and U24AG092760 from the National Institute on Aging (NIA). She is one of the leaders of the Alzheimer's Prevention Initiative (API), which is collaborating with Eli Lilly and Roche in its ongoing and planned AD prevention trials. She received consulting fees from Denovo Biopharma and Premiere Inc. N.J.A. received consultancy or speaker fees from Abbvie, Athria, ImaginationLand LLC, MapLight Therapeutics, SpearBio, Neurogen Biomarking, Quanterix, TauRx, Eli Lilly, Roche Dx, Beckman Coulter, Janssen, Bristol Myers Squibb, ImmunoBrain, Alamar Biosciences, Biogen, VJDementia; participates in Advisory Board for Biogen, Bristol Myers Squibb, New Amsterdam, Janssen, Roche and TauRx; and has patents planned, issued or pending Application No.: PCT/US2024/037834 (WSGR Docket No. 58484‐709.601) Methods for Remote Blood Collection, Extraction and Analysis of Neuro Biomarkers. EMR is supported by grants R01 AG058468, R01 AG086363, R01 AG055444, P30 AG072980, P30 AG019620, R01 AG069453, R01 NS139383, OT2 D026549, OT2 OD037642, T2 HL161847, P01 AG052350, R01 AG070883, RF1 AG073424, U24 AG072122, and R01 AG05467 from the National Institutes of Health, as well as by the JTMF Foundation, GHR Foundation, NOMIS Foundation, Banner Alzheimer's Foundation, Gates Ventures, and the Arizona Department of Health Services. He has received consulting fees and/or stock options as a scientific advisor to Alzheon, Beren Therapeutics, Denali, Enigma, Jocasta Neuroscience, and Vaxxinity. He is the inventor of a 2005 Banner Health patent concerning the use of biomarker endpoints to accelerate Alzheimer's prevention trials in cognitively unimpaired individuals at genetic or biomarker risk; this patent will not be deployed. He serves as Board Chair of the Flinn Foundation and the Arizona Alzheimer's Consortium and holds stock as a co‐founder, advisor, and shareholder of ALZPath, developer of a capture antibody licensed to several diagnostics companies for plasma p‐tau217 assays. All other authors (V.G., M.N.D., A.S., T.M.M., M.M.‐A., K.V.‐K.J., H.D.P., J.S., D.D.G., V.D., Y.C., S.L., Y.S.) report no conflict of interest. Author disclosures are available in the Supporting Information.

## CONSENT STATEMENT

Data used in this study were obtained from the Alzheimer's Disease Neuroimaging Initiative (ADNI). Written informed consent was obtained from all ADNI participants or their authorized representatives at each participating site, and ADNI protocols were approved by the institutional review boards of all participating institutions. The present study used de‐identified ADNI data.

## Supporting information




Supporting Information

Supporting Information
