# Supplementary material for: Prognostic value of plasma brain‐derived phosphorylated Tau
Source: Alzheimers Dement. 2026 Nov 13;22(11):e71983. doi: 10.1002/alz.71983 (PMC13768479; doi:10.1002/alz.71983)
Supplement: Supplementary file 2 [file ALZ-22-e71983-s001.docx]

Supplementary Table S1. Sensitivity analyses of plasma biomarker prediction of amyloid PET progression across alternative Centiloid thresholds

| **Marker** | **CL**  **threshold** | **tdAUC (95% CI) 2.5y** | **HR (95% CI)** | **Log-rank p** | **C-index** | **tdAUC (95% CI) 4y** | **HR (95% CI)** | **Log-rank p** | **C-index** |
| --- | --- | --- | --- | --- | --- | --- | --- | --- | --- |
| BD-pTau217 | 18 | 0.68 (0.51-0.85) | 2.03 (0.57-7.27) | 0.268 | 0.57 | 0.68 (0.51-0.85) | 2.03 (0.57-7.27) | 0.268 | 0.57 |
| pTau217 | 18 | 0.69 (0.54-0.85) | 2.53 (0.57-11.32) | 0.207 | 0.57 | 0.69 (0.53-0.84) | 2.53 (0.57-11.32) | 0.207 | 0.57 |
| BD-pTau181 | 18 | 0.58 (0.38-0.78) | 1.42 (0.48-4.23) | 0.529 | 0.53 | 0.57 (0.37-0.78) | 1.42 (0.48-4.23) | 0.529 | 0.53 |
| pTau181 | 18 | 0.60 (0.44-0.77) | 2.13 (0.67-6.79) | 0.192 | 0.58 | 0.60 (0.43-0.76) | 2.13 (0.67-6.79) | 0.192 | 0.58 |
| BD-pTau231 | 18 | 0.67 (0.52-0.82) | 5.56 (0.73-42.54) | 0.062 | 0.61 | 0.66 (0.51-0.81) | 5.56 (0.73-42.54) | 0.062 | 0.61 |
| pTau231 | 18 | 0.69 (0.53-0.85) | 4.36 (1.22-15.63) | 0.014 | 0.66 | 0.68 (0.52-0.84) | 4.36 (1.22-15.63) | 0.014 | 0.66 |
| GFAP | 18 | 0.56 (0.41-0.71) | 1.95 (0.65-5.83) | 0.223 | 0.57 | 0.55 (0.40-0.71) | 1.95 (0.65-5.83) | 0.223 | 0.57 |
| BD-pTau217 | 24.1 | 0.82 (0.72-0.92) | 10.54 (2.31-48.10) | <0.001 | 0.76 | 0.77 (0.65-0.90) | 7.03 (1.94-25.56) | <0.001 | 0.73 |
| pTau217 | 24.1 | 0.69 (0.53-0.85) | 2.15 (0.47-9.79) | 0.313 | 0.57 | 0.64 (0.47-0.81) | 1.42 (0.39-5.17) | 0.589 | 0.54 |
| BD-pTau181 | 24.1 | 0.66 (0.47-0.85) | 2.33 (0.63-8.60) | 0.192 | 0.59 | 0.60 (0.40-0.80) | 1.75 (0.54-5.69) | 0.345 | 0.57 |
| pTau181 | 24.1 | 0.55 (0.37-0.72) | 1.77 (0.56-5.58) | 0.322 | 0.57 | 0.50 (0.32-0.68) | 1.55 (0.51-4.72) | 0.442 | 0.55 |
| BD-pTau231 | 24.1 | 0.64 (0.47-0.81) | 3.48 (1.05-11.56) | 0.030 | 0.65 | 0.60 (0.44-0.77) | 2.78 (0.91-8.51) | 0.061 | 0.63 |
| pTau231 | 24.1 | 0.55 (0.37-0.72) | 1.87 (0.59-5.90) | 0.276 | 0.58 | 0.50 (0.32-0.67) | 1.55 (0.52-4.63) | 0.424 | 0.56 |
| GFAP | 24.1 | 0.66 (0.53-0.79) | 3.37 (0.91-12.44) | 0.053 | 0.64 | 0.68 (0.55-0.80) | 3.74 (1.03-13.61) | 0.031 | 0.65 |
| BD-pTau217 | 37 | 0.84 (0.75-0.93) | 10.84 (2.98-39.40) | <0.001 | 0.77 | 0.84 (0.75-0.93) | 10.84 (2.98-39.40) | <0.001 | 0.77 |
| pTau217 | 37 | 0.76 (0.63-0.89) | 8.93 (2.46-32.45) | <0.001 | 0.75 | 0.76 (0.62-0.89) | 8.93 (2.46-32.45) | <0.001 | 0.75 |
| BD-pTau181 | 37 | 0.74 (0.58-0.91) | 4.64 (1.28-16.85) | 0.010 | 0.68 | 0.74 (0.57-0.90) | 4.64 (1.28-16.85) | 0.010 | 0.68 |
| pTau181 | 37 | 0.60 (0.44-0.75) | 2.80 (0.94-8.33) | 0.054 | 0.62 | 0.59 (0.44-0.75) | 2.80 (0.94-8.33) | 0.054 | 0.62 |
| BD-pTau231 | 37 | 0.70 (0.52-0.88) | 7.31 (2.01-26.60) | <0.001 | 0.73 | 0.69 (0.51-0.88) | 7.31 (2.01-26.60) | <0.001 | 0.73 |
| pTau231 | 37 | 0.64 (0.47-0.80) | 4.21 (1.16-15.31) | 0.017 | 0.67 | 0.63 (0.46-0.80) | 4.21 (1.16-15.31) | 0.017 | 0.67 |
| GFAP | 37 | 0.70 (0.57-0.82) | 3.93 (1.32-11.70) | 0.008 | 0.66 | 0.69 (0.57-0.82) | 3.93 (1.32-11.70) | 0.008 | 0.66 |
| Sensitivity analyses evaluating prognostic performance of individual plasma biomarkers for prediction of amyloid PET progression (A− to A+) among baseline cognitively unimpaired participants using alternative amyloid positivity thresholds (Centiloid ≥18, ≥24.1, and ≥37). Discrimination is summarized using time-dependent area under the curve (tdAUC) at prespecified 2.5- and 4-year horizons. Hazard ratios (HRs) and C-indices were derived from Cox proportional hazards models comparing high versus low biomarker groups within each horizon. Log-rank p-values compare Kaplan-Meier curves for the same high versus low biomarker groups. HRs compare high versus low biomarker groups defined by Youden cutoffs within each threshold. CL indicates Centiloid. | | | | | | | | | |

Supplementary Table S2. Prediction of amyloid PET progression using plasma pTau/Aβ42 ratio measures and additional pTau species

| **Marker** | **CL**  **threshold** | **tdAUC (95% CI)**  **2.5y** | **HR (95% CI)** | **Log-rank p** | **C-index** | **tdAUC (95% CI)**  **4y** | **HR (95% CI)** | **Log-rank p** | **C-index** |
| --- | --- | --- | --- | --- | --- | --- | --- | --- | --- |
| BD-pTau217/Aβ42 | 18 | 0.70 (0.56–0.85) | 5.14 (1.72–15.33) | 0.001 | 0.69 | 0.70 (0.56–0.84) | 5.14 (1.72–15.33) | 0.001 | 0.69 |
| pTau217/Aβ42 | 18 | 0.71 (0.56–0.87) | 5.74 (1.60–20.59) | 0.002 | 0.69 | 0.70 (0.55–0.86) | 5.74 (1.60–20.59) | 0.002 | 0.69 |
| BD-pTau181/Aβ42 | 18 | 0.64 (0.45–0.82) | 3.90 (1.31–11.64) | 0.008 | 0.65 | 0.63 (0.45–0.81) | 3.90 (1.31–11.64) | 0.008 | 0.65 |
| pTau181/Aβ42 | 18 | 0.62 (0.44–0.79) | 2.08 (0.47–9.31) | 0.325 | 0.56 | 0.61 (0.44–0.79) | 2.08 (0.47–9.31) | 0.325 | 0.56 |
| BD-pTau231/Aβ42 | 18 | 0.67 (0.53–0.81) | 5.59 (1.56–20.05) | 0.003 | 0.69 | 0.67 (0.52–0.81) | 5.59 (1.56–20.05) | 0.003 | 0.69 |
| pTau231/Aβ42 | 18 | 0.67 (0.51–0.84) | 3.34 (0.93–11.97) | 0.049 | 0.63 | 0.67 (0.50–0.83) | 3.34 (0.93–11.97) | 0.049 | 0.63 |
| Aβ40/Aβ42 | 18 | 0.53 (0.35–0.71) | 2.89 (1.00–8.32) | 0.04 | 0.62 | 0.54 (0.36–0.72) | 2.89 (1.00–8.32) | 0.04 | 0.62 |
| BD-pTau217/Aβ42 | 24.1 | 0.59 (0.43–0.74) | 2.37 (0.76–7.34) | 0.124 | 0.61 | 0.58 (0.44–0.72) | 2.02 (0.68–6.01) | 0.197 | 0.59 |
| pTau217/Aβ42 | 24.1 | 0.60 (0.44–0.76) | 3.12 (0.94–10.38) | 0.05 | 0.64 | 0.58 (0.43–0.73) | 2.49 (0.81–7.61) | 0.098 | 0.62 |
| BD-pTau181/Aβ42 | 24.1 | 0.55 (0.35–0.75) | 2.93 (0.93–9.25) | 0.054 | 0.63 | 0.52 (0.34–0.71) | 2.44 (0.82–7.25) | 0.098 | 0.61 |
| pTau181/Aβ42 | 24.1 | 0.50 (0.34–0.66) | 1.80 (0.39–8.19) | 0.444 | 0.55 | 0.48 (0.32–0.63) | 1.96 (0.44–8.86) | 0.371 | 0.55 |
| BD-pTau231/Aβ42 | 24.1 | 0.43 (0.27–0.59) | 1.08 (0.35–3.34) | 0.897 | 0.51 | 0.45 (0.30–0.60) | 1.25 (0.42–3.73) | 0.685 | 0.52 |
| pTau231/Aβ42 | 24.1 | 0.49 (0.32–0.66) | 1.65 (0.53–5.12) | 0.379 | 0.56 | 0.46 (0.30–0.62) | 1.42 (0.48–4.21) | 0.53 | 0.55 |
| Aβ40/Aβ42 | 24.1 | 0.44 (0.27–0.61) | 0.74 (0.16–3.37) | 0.695 | 0.52 | 0.48 (0.31–0.65) | 1.10 (0.30–4.00) | 0.883 | 0.5 |
| BD-pTau217/Aβ42 | 37 | 0.75 (0.64–0.87) | 5.68 (1.75–18.46) | 0.001 | 0.71 | 0.75 (0.64–0.87) | 5.68 (1.75–18.46) | 0.001 | 0.71 |
| pTau217/Aβ42 | 37 | 0.76 (0.66–0.87) | 8.15 (1.81–36.77) | 0.001 | 0.73 | 0.76 (0.65–0.86) | 8.15 (1.81–36.77) | 0.001 | 0.73 |
| BD-pTau181/Aβ42 | 37 | 0.76 (0.64–0.88) | 10.71 (2.37–48.34) | <0.001 | 0.76 | 0.75 (0.64–0.87) | 10.71 (2.37–48.34) | <0.001 | 0.76 |
| pTau181/Aβ42 | 37 | 0.64 (0.52–0.77) | NE (separation) | 0.034 | 0.63 | 0.64 (0.52–0.77) | NE (separation) | 0.034 | 0.63 |
| BD-pTau231/Aβ42 | 37 | 0.61 (0.45–0.77) | 1.94 (0.65–5.78) | 0.224 | 0.58 | 0.61 (0.45–0.76) | 1.94 (0.65–5.78) | 0.224 | 0.58 |
| pTau231/Aβ42 | 37 | 0.67 (0.56–0.79) | 10.64 (1.38–81.83) | 0.005 | 0.7 | 0.67 (0.55–0.78) | 10.64 (1.38–81.83) | 0.005 | 0.7 |
| Aβ40/Aβ42 | 37 | 0.52 (0.33–0.72) | 3.77 (1.04–13.70) | 0.03 | 0.58 | 0.53 (0.33–0.72) | 3.77 (1.04–13.70) | 0.03 | 0.58 |
| Time-dependent discrimination and risk estimates for prediction of amyloid PET progression (A− to A+) among baseline cognitively unimpaired participants. Results are shown for plasma pTau/Aβ42 ratio measures and additional phosphorylated tau species across multiple amyloid positivity thresholds (CL ≥18, ≥24.1, and ≥37). Discrimination is summarized using time-dependent area under the curve (tdAUC) at prespecified 2.5- and 4-year horizons. Hazard ratios (HRs) and C-indices were derived from Cox proportional hazards models comparing high versus low biomarker groups within each horizon. Log-rank p-values compare Kaplan-Meier (KM) curves for the same high versus low biomarker groups. HRs compare high versus low biomarker groups defined by Youden cutoffs within each threshold. In some threshold-specific analyses, estimates were the same at 2.5 and 4 years because all amyloid conversion events observed within the 4-year horizon occurred within the first 2.5 years. CL indicates Centiloid. NE indicates that the Cox hazard ratio could not be estimated because no progression events occurred in one biomarker group, resulting in complete separation. The log-rank p-value remains estimable because it tests differences between the survival curves without estimating an HR; the C-index summarizes rank discrimination and does not require an HR. | | | | | | | | | |
